# Lie symmetry analysis and solitary wave solution of biofilm model Allen-Cahn

**DOI:** 10.1038/s41598-024-62315-5

**Published:** 2024-06-04

**Authors:** Muhammad Shakeel, Naseem Abbas, Muhammad Junaid U. Rehman, Fehaid Salem Alshammari, Abdullah Al-Yaari

**Affiliations:** 1 School of Mathematics and Statistics, Central South University, Changsha, 410083 China; 2 Department of Mathematics, Quaid-e-Azam University 45320, Islamabad, 44000 Pakistan; 3 Department of Automation, Biomechanics, and Mechatronics, Lodz University of Technology, 1/15 Stefanowski St. (Building A22), Lodz, 90-924 Poland; 4 Department of Mathematics and Statistics, College of Science, Imam Mohammad Ibn Saud Islamic University (IMSIU), Riyadh, Saudi Arabia; 5 Department of Mathematics, Faculty of Applied Science, Thamar University, Dhamar, 00967 Yemen

**Keywords:** Biofilm model, Lie symmetry technique, Solitory wave solution, Engineering, Mathematics and computing

## Abstract

The investigation presented in this study delves into the analysis of Lie symmetries for the bistable Allen-Cahn (BAC) equation with a quartic potential, specifically applied to the biofilm model. By employing the Lie symmetry method, we have acquired the Lie infinitesimal generators for the considered model. Using a transformation method, the nonlinear partial differential equations (NPDEs) are converted into various nonlinear ordinary differential equations (NLODEs), providing the numerous closed-form solitary wave solutions. The obtained solutions manifest in various forms including dark, bright, kink, anti-kink, and periodic types using diverse strategies. To enhance the physical interpretation, the study presents 3D, 2D, and contour plots of the acquired solutions. Every graph’s wave-like structure contains information about the structural behaviour of the bacteria that build biofilms on surfaces where rectangles have different densities. This analysis enhances comprehension of the complex dynamics present in areas like fluid dynamics, fiber optics, biology, ocean physics, coastal engineering, and nonlinear complex physical systems.

## Introduction

PDEs has main contributions to the natural world are as follows, fluid dynamics, vibration of solids, propagation of sound, the phenomenon of electromagnetic radiation, optics, plasma, heat flow and diffusion, and structural properties of molecules with the interaction between photons and electrons^1–3^. In recent mathematics, PDEs are essential to the study of infinite-dimensional group representation, homogeneous spaces, mathematical physics, and quantum field theory. PDEs are used as a link between physical science and applied mathematics. Our primary focus is the solitary wave solutions of nonlinear PDEs in term of biofilm model. The spatial structure, dynamics, and stability of microbial communities within biofilms are significantly shaped by these solitary wave occurrences. They support the development of intricate spatial patterns, the spread of ecological disturbances, and the adaptability of biofilm ecosystems to changing environmental conditions. With implications for a variety of applications, including bioremediation, biofilm engineering, and medical microbiology, an understanding of the mechanisms driving solitary wave occurrences in biofilm models offers insights into microbial community dynamics, spatial patterning, and ecosystem function. The characteristics of nonlinear PDEs are related to the nonlinear natural laws. They are employed in various fields, including stochastic game theory, fluid dynamics, gravitational behavior, the Poincaré and Calabi conjectures, non-Newtonian fluid, etc. Nonlinear PDE solutions can be found using a variety of methods. Iqbal et al. find the various types of travelling solitary wave solutions of stochastic AC equation by extended fan-sub tech, whereas Jornet considers biofilm formation via the stochastic BAC equation^4^, Tijani analyze the biofilm formation of AC equation by utilizing finite difference scheme^5^, and also Tijani apply the finite difference technique to biofilm growth model^6^. Finding soliton solutions is a crucial area of research in the current era to characterize the physical behavior of nonlinear PDEs. Many efficient techniques have been utilized to analyze the solutions of NPDEs, such as the Ricatti equation approach^7^, the Kudryashov scheme^8^, Darboux approach^9^, the Jacobi elliptic function technique^10^, the extended sinh-Gordon technique^11^, the direct algebraic scheme^12^, the extended tanh technique^13^, Fokas method^14^, the Hirota bilinear method^15^, the first integral approach^16^, the trial solution approach^17^, the $\left( \frac{G^{'}}{G}\right)$-expansion technique^18,19^, Inverse $\left( \frac{G^{'}}{G}\right)$-expansion technique^20^, the $\left( \frac{G^{'}}{G^2}\right)$-expansion approach^21^, the modified auxiliary equation method^15^, the Lie symmetry technique^22,23^, the unified approach^24^, and so on.

It has encountered extensive application of the proposed model in the exploration of various physical problems, including crystal growth, image segmentation, and motion by mean curvature flows. Particularly, it serves as a fundamental model equation within the diffuse interface approach, which is instrumental in studying phase transitions and interfacial dynamics in material science. Therefore, the quest for an efficient and precise method to solve this equation maintains practical importance and has garnered significant attention from researchers. Despite the challenges posed by nonlinear and stiff differential equations, improvements in modern computing technology and sophisticated software have facilitated the exploration of approximate analytical solutions for such problems.

Aside from numerical computation, symmetry analysis employing Lie group theory is the most significant technique for tackling nonlinear issues^25–29^. Almost any system of differential equations can have its symmetries found utilizing this method, and understanding these symmetries can make it easier to analyse physical issues that the equations regulate. The simple way to reduce similarity is to utilize the non-classical symmetry^30^ or Lie symmetry group^31,32^ techniques. The deciding systems for reducing non-autonomous NPDEs to NODEs are algebraic equations; however, if this reduction cannot be performed directly, alternative strategies must be taken into account. This article deals with the nonlinear PDE in the kind of a biofilm model. We used the generalized auxiliary equation method, modified $\left( \frac{G^{'}}{G^2}\right)$-expansion method and extended the modified tanh expansion scheme to find exact soliton solutions of the biofilm model. The dynamical nature of these methods illuminates the soliton solutions as well as the hyperbolic and trigonometric function solutions; see^33,34^. One straightforward illustration of a biofilm is the plague coating that typically develops on teeth.

A biofilm can be understood as a conglomerate of one or multiple species of microorganisms that adhere to diverse surfaces. These microorganisms encompass bacteria, fungi, and algae^4,35^. Thus, these findings are novel and have an impact on the biofilm-forming colonies of living, reproducing bacteria. The bistable Allen-Cahn equation (with quartic potential) is the nonlinear dynamical biofilm model that we take into consideration in this paper^36^

1 $$\begin{aligned} \mathcal {M}_{t}-d\mathcal {M}_{xx}-d \mathcal {M}_{yy}+\gamma \mathcal {M}^3-\gamma (1+\sigma )\mathcal {M}^2+\gamma \sigma \mathcal {M}=0, \end{aligned}$$

where $\mathcal {M}(t, x, y)$ is the density of Microbes on the rectangular surface $[e, g]\times [n, m]$ (i.e., $(x, y)\in [e, g]\times [n, m]$, and time $t\ne 0$). $d>0$ is the diffusion coefficient, $\gamma >0$ is the growth rate, the parameter $\sigma \in [0, 1]$ encodes the survival rate of Microbes^4^. The present article is divided into different sections. The symmetry analysis technique is presented in Sect. "Lie point symmetries of Equation (1)". Sections "Methodologies" and "Solitary wave solutions" for methodologies and solitary wave solutions. The different kinds of soliton are discussed in Sect. "Graphical discussion". In the end, the conclusion is present in Sect. "Conclusion".

## Lie point symmetries of Eq. (1)

This section will use the Lie symmetry technique for the understudy Eq. (1). Now, consider the one-parameter Lie group of infinitesimal transformations on $(t,x,y,\mathcal {M})$ given by:

$$\begin{aligned} \bar{t}=&t+\varepsilon ~\chi ^{1}(t,x,y,\mathcal {M})+O(\varepsilon ^{2}),\\ \bar{x}=&x+\varepsilon ~\chi ^{2}(t,x,y,\mathcal {M})+O(\varepsilon ^{2}),\\ \bar{y}=&y+\varepsilon ~\chi ^{3}(t,x,y,\mathcal {M})+O(\varepsilon ^{2}),\\ \bar{M}=&M+\varepsilon ~\chi ^{4}(t,x,y,\mathcal {M})+O(\varepsilon ^{2}), \end{aligned}$$

When a group parameter $\varepsilon \ll 1$ is utilized. Vector fields span the corresponding Lie algebra of infinitesimal symmetries.

2 $$\begin{aligned} \mathcal {X}=\chi ^{1} (t,x,y,\mathcal {M})\frac{\partial }{\partial {t}} +\chi ^2(t,x,y,\mathcal {M}) \frac{\partial }{\partial {x}}&+\chi ^{3} (t,x,y,\mathcal {M}) \frac{\partial }{\partial {y}}+\phi (t,x,y,\mathcal {M}) \frac{\partial }{\partial {\mathcal {M}}}\cdot \end{aligned}$$

This vector field (2) generates a symmetry of Eq. (1), and satisfies the Lie in-variance condition as

$$\begin{aligned} \mathcal {X}^{(2)}(\mathcal {M}_{t}-d\mathcal {M}_{xx}-d \mathcal {M}_{yy}+\gamma \mathcal {M}^3-\gamma (1+\sigma )\mathcal {M}^2+\gamma \sigma \mathcal {M})\big |_{Eq.(1)}=0, \end{aligned}$$

where $\mathcal {X}^{(2)}$ is the second prolongation of $\mathcal {X}$ is written as:

3 $$\begin{aligned} \mathcal {X}^{(2)}=&\mathcal {X}+\phi ^{t}\frac{\partial }{\partial \mathcal {M}_{t}}+\phi ^{x x}\frac{\partial }{\partial \mathcal {M}_{xx}}+\phi ^{yy}\frac{\partial }{\partial \mathcal {M}_{y y}}\cdot \end{aligned}$$

Furthermore, we have:

4 $$\begin{aligned} {\left\{ \begin{array}{ll} \phi ^{t}=D_{t}(\phi )-\mathcal {M}_{t}D_{t}(\chi ^{1})-\mathcal {M}_{x}D_{t}(\chi ^{2}) -\mathcal {M}_{y}D_{t}(\chi ^{3}),\\ \phi ^{x}=D_{x}(\phi )-\mathcal {M}_{t}D_{x}(\chi ^{1})-\mathcal {M}_{x}D_{x}(\chi ^{2}) -\mathcal {M}_{y}D_{x}(\chi ^{3}),\\ \phi ^{y}=D_{y}(\phi )-\mathcal {M}_{t}D_{y}(\chi ^{1})-\mathcal {M}_{x}D_{y}(\chi ^{2}) -\mathcal {M}_{y}D_{y}(\chi ^{3}),\\ \phi ^{y y}=D_{y}(\phi ^{y})-\mathcal {M}_{t y}D_{y}(\chi ^{1})-\mathcal {M}_{x t}D_{y}(\chi ^{2})-\mathcal {M}_{y y}D_{y}(\chi ^{3}),\\ \phi ^{x x}=D_{x}(\phi ^{x})-\mathcal {M}_{x x}D_{x}(\chi ^{1})-\mathcal {M}_{x x}D_{x}(\chi ^{2})-\mathcal {M}_{x x}D_{x}(\chi ^{3}). \end{array}\right. } \end{aligned}$$

Applying in-variance condition to Eq. (1), we have

5 $$\begin{aligned} \big (3\gamma \mathcal {M}^2-2\gamma (1+\sigma )\mathcal {M}+\gamma \sigma +\phi ^{t}-d\phi ^{xx}-d\phi ^{yy}\big )\big |_{Eq.(1)}=0, \end{aligned}$$

putting the values of $\phi ^i$ from the (4), we obtain an equation in partial derivatives of $\chi ^1$, $\chi ^2$, $\chi ^3$ and $\phi$. Equating by using *Maple* we have

6 $$\begin{aligned} \chi ^1=&\mathcal {C}_3,\\ \chi ^2=&-\mathcal {C}_1y+\mathcal {C}_4,\\ \chi ^3=&\mathcal {C}_{1}x+\mathcal {C}_2,\\ \phi =&0, \end{aligned}$$

where $\mathcal {C}_{i},~i=1,2,3,4$ are arbitrary constants.

As a result, (1) symmetries have the following structure:

7 $$\begin{aligned} \mathcal {X}_{1}=&\frac{\partial }{\partial t},\\ \mathcal {X}_{2}=&\frac{\partial }{\partial x},\\ \mathcal {X}_{3}=&\frac{\partial }{\partial y},\\ \mathcal {X}_{4}=&y\frac{\partial }{\partial x}-x\frac{\partial }{\partial y}\cdot \end{aligned}$$

The above table makes it evident that a 4-dimensional Lie algebra is formed by all of the generators of Eq. (1) that were developed. Next, $\mathcal {X}_{i}$’s linear combination is provided as follows:

8 $$\begin{aligned} {\mathcal {X}}=\mathcal {C}_{1}{\mathcal {X}}_{1}+\mathcal {C}_{2}{\mathcal {X}}_{2}+\mathcal {C}_{3}{\mathcal {X}}_{3}+\mathcal {C}_{4}{\mathcal {X}}_{4}. \end{aligned}$$

The vector fields give commutation relations for Eq. 1, (*i*, *j*)-th entry of Table 1 is the Lie bracket $[{\mathcal {X}}_{i},{\mathcal {X}}_{j}]={\mathcal {X}}_{i}{\mathcal {X}}_{j}-{\mathcal {X}}_{j}{\mathcal {X}}_{i}$, where $i,j=1,2,3,4$. We noted that Table 1 is skew-symmetric with zero diagonal elements and it shows that the generators ${\mathcal {X}}_{i}$, $1\le i \le 4$ are linearly independent.

**Table 1:** Commutator table of Lie Algebra.

$[{\mathcal {X}}_{i},{\mathcal {X}}_{j}]$	${\mathcal {X}}_{1}$	${\mathcal {X}}_{2}$	${\mathcal {X}}_{3}$	${\mathcal {X}}_{4}$
${\mathcal {X}}_{1}$	0	0	0	0
${\mathcal {X}}_{2}$	0	0	0	$-{\mathcal {X}}_{3}$
${\mathcal {X}}_{3}$	0	0	0	${\mathcal {X}}_{2}$
${\mathcal {X}}_{4}$	0	${\mathcal {X}}_{3}$	$-{\mathcal {X}}_{2}$	0

### Reductions using symmetries

Using similarity variables, we will find reduction equations in this section. After solving reduction equations we can achieved the solitary wave solutions.

**Case 1:** The characteristic equations for the infinitesimal generator are provided by $\mathcal {X}_{1}=\frac{\partial }{\partial t}$,

9 $$\begin{aligned} \frac{dt}{1}=\frac{dx}{0}=\frac{dy}{0}=\frac{dM}{0}\cdot \end{aligned}$$

The group invariant solution $\mathcal {M}=f(X,Y)$ is found after solving the characteristic equation above with the similarity variables $X=x$ and $Y=y$. The value of $\mathcal {M}$ is then entered into (1), and the following reduction equation is calculated:

10 $$\begin{aligned} -d f_{xx}-d f_{yy}+\gamma f^3-\gamma (1+\sigma )f^2+\gamma \sigma f=0. \end{aligned}$$

Utilizing the similarity transformation technique once more on (10), we discover a fresh collection of infinitesimals denoted as

11 $$\begin{aligned} \eta _{f}=0,~~\xi _{x}=-C_{1}y+C_3,~~\xi _{y}=C_{1}x+C_2. \end{aligned}$$

If $C_1=C_2=C_3=1$, then f can be written as:

12 $$\begin{aligned} f=u(r), ~~~ r=x^2+2x-2y+y^2. \end{aligned}$$

From Eqs. (10) and (12), we get the following equation,

13 $$\begin{aligned} 4d u^{''}-\gamma u^3+\gamma (1+\sigma )u^2-\gamma \sigma u=0. \end{aligned}$$

**Case 2:** The characteristic equations for the infinitesimal generator are provided by $\mathcal {X}_{2}=\frac{\partial }{\partial x}$,

14 $$\begin{aligned} \frac{dt}{0}=\frac{dx}{1}=\frac{dy}{0}=\frac{dM}{0}\cdot \end{aligned}$$

The similarity variables are $Y=y, T=t$ and the group invariant solution is $\mathcal {M}=f(Y,T)$, substituting the value of $\mathcal {M}$ in (1), we attain the reduction equation as follows:

15 $$\begin{aligned} f_{t}-d f_{yy}+\gamma f^3-\gamma (1+\sigma )f^2+\gamma \sigma f=0. \end{aligned}$$

Utilizing the similarity transformation technique once more on (15), we discover a fresh collection of infinitesimals denoted as

16 $$\begin{aligned} \eta _{f}=0,~~\xi _{t}=C_{1},~~\xi _{y}=C_2. \end{aligned}$$

If $C_1=C_2=1$ then following form *f* can be written as

17 $$\begin{aligned} f=u(t-y),~~t-y=r. \end{aligned}$$

From equations (10) and (12), we get the following equation,

18 $$\begin{aligned} u^{'}-d u^{''}+\gamma u^3-\gamma (1+\sigma )u^2+\gamma \sigma u=0. \end{aligned}$$

**Case 3:** The characteristic equations for the infinitesimal generator are provided by $\mathcal {X}_{3}=\frac{\partial }{\partial y}$,

19 $$\begin{aligned} \frac{dt}{0}=\frac{dx}{0}=\frac{dy}{1}=\frac{dM}{0}\cdot \end{aligned}$$

The similarity variables are $X=x, T=t$ and the group invariant solution is $\mathcal {M}=f(X,T)$, substituting the value of $\mathcal {M}$ in (1), we achieved the reduction equation as follows:

20 $$\begin{aligned} f_{t}-d f_{xx}+\gamma f^3-\gamma (1+\sigma )f^2+\gamma \sigma f=0. \end{aligned}$$

Again applying the similarity transformation technique on (20), we can determine the following new set of infinitesimals:

21 $$\begin{aligned} \eta _{f}=0,~~\xi _{t}=C_{1},~~\xi _{x}=C_2. \end{aligned}$$

If $C_1=C_2=1$ then value of *f* can be written as

22 $$\begin{aligned} f=u(t-x),~~t-x=r. \end{aligned}$$

From Eqs. (10) and (12), we get the following equation,

23 $$\begin{aligned} u^{'}-d u^{''}+\gamma u^3-\gamma (1+\sigma )u^2+\gamma \sigma u=0. \end{aligned}$$

**Case 4:** The characteristic equations for the infinitesimal generator are provided by $\mathcal {X}_{4}=y\frac{\partial }{\partial x}-x\frac{\partial }{\partial y}$.

24 $$\begin{aligned} \frac{dt}{0}=\frac{dx}{y}=\frac{dy}{-x}=\frac{dM}{0}\cdot \end{aligned}$$

The group invariant solution is $\mathcal {M}=f(X,Y)$, and the similarity variables are $X=y,~Y=-x$. By substituting the value of $\mathcal {M}$ in Eq. (1), we were able to construct the reduction equation as follows:

25 $$\begin{aligned} f_{t}-2d f_{xx}-2d f_{yy}+\gamma f^3-\gamma (1+\sigma )f^2+\gamma \sigma f=0. \end{aligned}$$

Utilizing the similarity transformation technique once more on (25), we discover a fresh collection of infinitesimals denoted as

26 $$\begin{aligned} \eta _{f}=0,~~\xi _{t}=C_{1},~~\xi _{x}=C_2. \end{aligned}$$

If $C_1=C_2=1$ then following form *f* can be written as

27 $$\begin{aligned} f=u(t-x),~~t-x=r. \end{aligned}$$

From Eqs. (10) and (12), we get the following equation

28 $$\begin{aligned} u^{'}-2d u^{''}+\gamma u^3-\gamma (1+\sigma )u^2+\gamma \sigma u=0. \end{aligned}$$

## Methodologies

### Generalized auxiliary equation method

Let the NPDE is

29 $$\begin{aligned} Q\left( f, \frac{\partial f}{\partial y}, \frac{\partial f}{\partial t}, \cdots \right) . \end{aligned}$$

Suppose the Eq. (29) can be changed into nonlinear ordinary differential equation (NODE) as:

30 $$\begin{aligned} O(u, u^{'}, u^{''}\cdots )=0. \end{aligned}$$

Let us suppose that Eq. (30) has solutions as follows,

31 $$\begin{aligned} u(r)=a_{0}+\sum _{i=0}^{N}a_i \varsigma (r), ~~ a_{N}\ne 0 \end{aligned}$$

where $a_i(i=1,2,3,\cdot ,N)$, are the constant to be determined and we use the balance method to find the value of *N*. The function $\varsigma )$ satisfies the auxiliary equation defined as:

32 $$\begin{aligned} r^{'}(\varsigma )=\sqrt{b_{1}\varsigma ^{3}(r)+b_{2}\varsigma ^{3}(r)+b_{3}\varsigma ^{4}(r)}. \end{aligned}$$

where $b_{m},~m=1,2,3$ are real constants. The following are the solutions set of Eq. (32).

#### Hyperbolic trigonometric solutions

33 $$\begin{aligned} & \varsigma (r)=\frac{-b_{1}b_{2}\textrm{sech} \,^{2}\left( \frac{\sqrt{b_{1}}}{2}r\right) }{b^{2}_{2}-b_{1}b_{3}\left( 1\pm \tanh \left( \frac{\sqrt{b_{1}}}{2}r\right) \right) },~b_{1}>0. \end{aligned}$$

34 $$\begin{aligned} & \varsigma (r)=\frac{b_{1}b_{2}\textrm{csch} \,^{2}\left( \frac{\sqrt{b_{1}}}{2}r\right) }{b^{2}_{2}-b_{1}b_{3}\left( 1\pm \coth \left( \frac{\sqrt{b_{1}}}{2}r\right) \right) },~b_{1}>0. \end{aligned}$$

35 $$\begin{aligned} & \varsigma (r)=\frac{2b_{1}\textrm{sech} \,^{2}\left( \sqrt{b_{1}}r\right) }{\pm \sqrt{\zeta }-b_{2}\textrm{sech} \,\left( \sqrt{b_{1}}r\right) },~b_{1}>0, \zeta >0. \end{aligned}$$

36 $$\begin{aligned} & \varsigma (r)=\frac{2b_{1}\textrm{csch} \,^{2}\left( \sqrt{b_{1}}r\right) }{\pm \sqrt{-\zeta }-b_{2}\textrm{sech} \,\left( \sqrt{b_{1}}r\right) },~b_{1}>0, \zeta >0. \end{aligned}$$

37 $$\begin{aligned} & \varsigma (r)=\frac{-b_{1}\textrm{sech} \,^{2}\left( \frac{\sqrt{b_{1}}}{2}r\right) }{b_{2}\pm 2\sqrt{b_{1}b_{3}}\tanh \left( \frac{\sqrt{b_{1}}}{2}r\right) },~b_{1}>0,b_{3}>0. \end{aligned}$$

38 $$\begin{aligned} & \varsigma (r)=\frac{b_{1}\textrm{csch} \,^{2}\left( \frac{\sqrt{b_{1}}}{2}r\right) }{b_{2}\pm 2\sqrt{b_{1}b_{3}}\coth \left( \frac{\sqrt{b_{1}}}{2}r\right) },~b_{1}>0,b_{3}>0. \end{aligned}$$

39 $$\begin{aligned} & \varsigma (r)=-\frac{b_{1}}{b_{2}}\left( 1\pm \tanh \left( \frac{\sqrt{b_{1}}}{2}r\right) \right) ,~b_{1}>0,~\zeta =0. \end{aligned}$$

40 $$\begin{aligned} & \varsigma (r)=-\frac{b_{1}}{b_{2}}\left( 1\pm \coth \left( \frac{\sqrt{b_{1}}}{2}r\right) \right) .~b_{1}>0.\zeta =0. \end{aligned}$$

#### Trigonometric solutions

41 $$\begin{aligned} &  \varsigma (r)=\frac{-b_{1}\sec ^{2}\left( \frac{\sqrt{-b_{1}}}{2}r\right) }{b_{2}\pm 2\sqrt{-b_{1}b_{3}}tan\left( \frac{\sqrt{-b_{1}}}{2}r\right) },~b_{1}<0,b_3>0. \end{aligned}$$

42 $$\begin{aligned} &  \varsigma (r)=\frac{-b_{1}\csc ^{2}\left( \frac{\sqrt{-b_{1}}}{2}r\right) }{b_{2}\pm 2\sqrt{-b_{1}b_{3}}\cot \left( \frac{\sqrt{-b_{1}}}{2}r\right) },~b_{1}<0,b_3>0. \end{aligned}$$

43 $$\begin{aligned} &  \varsigma (r)=\frac{2b_{1}\sec ^{2}\left( \sqrt{-b_{1}}r\right) }{\pm \sqrt{\zeta }-b_{2}\sec \left( \sqrt{-b_{1}}r\right) },~b_{1}<0, \zeta >0. \end{aligned}$$

44 $$\begin{aligned} &  \varsigma (r)=\frac{2b_{1}\csc ^{2}\left( \sqrt{-b_{1}}r\right) }{\pm \sqrt{\zeta }-b_{2}\sec \left( \sqrt{-b_{1}}r\right) }.~b_{1}<0, \zeta >0. \end{aligned}$$

#### Exponential solutions

45 $$\begin{aligned} &  \varsigma (r)=\frac{4b_1 e^{\pm \sqrt{b_1}r}}{\left( e^{\pm \sqrt{b_1}r}-b_2\right) ^2-4b_1b_3},~b_{1}>0. \end{aligned}$$

46 $$\begin{aligned} &  \varsigma (r)=\frac{\pm 4b_1 e^{\pm \sqrt{b_1}r}}{1-4b_1b_3e^{\pm 2\sqrt{b_1}r}},~b_{1}>0, b_2=0. \end{aligned}$$

#### Rational solutions

47 $$\begin{aligned} &  \varsigma (r)=\frac{\pm b_1b_2}{b_2^2r^2-b_1b_3},~b_1=0. \end{aligned}$$

48 $$\begin{aligned} &  \varsigma (r)=\pm \frac{1}{\sqrt{b_3}r},~b_1=0, b_2=0. \end{aligned}$$

where $\zeta =b_2^2-4b_1b_3$.. As Eqs.

Eq. (32) and Eq. (31) are substituted into Eq. (30) and then we get a set of an algebraic equation. Solving these equations then we get a solution of NPDE Eq. (29).

### The modified $\left( \frac{G^{'}}{G^2}\right)$-expansion method

The travelling wave solutions is

49 $$\begin{aligned} &  U(r)=\sum _{n=0}^{N}a_n(\frac{G^{'}}{G^2})^n. \end{aligned}$$

50 $$\begin{aligned} &  \left( \frac{G^{'}}{G^2}\right) ^{'}=\tau +\kappa \left( \frac{G^{'}}{G^2}\right) ^2, \end{aligned}$$

where $\tau \ne 0, \kappa \ne 1$ are integers $a_n$ are unknown constants which to be found later,

for $n=1,2,3,...N$. The Eq. (50) has three cases: **Case 1:** If $\kappa \tau >0$,

51 $$\begin{aligned} \left( \frac{G^{'}}{G^2}\right) =\sqrt{\frac{\kappa }{\tau }}\left( \frac{A_1\cos \sqrt{\kappa \tau }\rho +B_1\sin \sqrt{\kappa \tau }\rho }{A_1\sin \sqrt{\kappa \tau } \rho -B_1\cos \sqrt{\kappa \tau }\rho }\right) , \end{aligned}$$

where $A_1$ and $B_1$ are arbitrary nonzero constants.

**Case 2:** If $$\kappa \tau <0$$,

52 $$\begin{aligned} \left( \frac{G^{'}}{G^2}\right) =-\frac{\sqrt{\mid \kappa \tau \mid }}{\tau }+\frac{\sqrt{\mid \kappa \tau \mid }}{2}\left( \frac{A_1\sinh (2\sqrt{\mid \kappa \tau \mid }\rho )+B_1\cosh (2\sqrt{\mid \kappa \tau \mid }\rho )}{A_1 \cosh (2\sqrt{\mid \kappa \tau \mid }\rho )+B_1\sinh (2\sqrt{\mid \kappa \tau \mid }\rho )}\right) . \end{aligned}$$

**Case 3:** If $$\kappa =0, \tau \ne 0$$,

53 $$\begin{aligned} \left( \frac{G^{'}}{G^2}\right) =-\frac{A_1}{\tau (A_1\rho +B_1)}\cdot \end{aligned}$$

All three types of solutions can be obtained by putting the values of unknowns $a_0, a_n$, and the Eqs. (51-53) into Eq. (49).

### Extended modified $\tanh$ expansion scheme

Consider the solution of Eq. (29),

54 $$\begin{aligned} u(r)=a_0+\sum _{n=1}^N a_n \varsigma ^n(r) + \sum _{n=1}^N b_n \varsigma ^{-n}(r). \end{aligned}$$

In Eq. (54), $a_0, a_n$, and $b_n$ are all constants to be determined later. At the same time, both $a_n$ and $b_n$ are not zero. Where $(n=1,2,...,N)$, after this we apply the balance technique on Eq. (54), then we get the value of *N*.

Consider the equation

55 $$\begin{aligned} \varsigma '(r)=\kappa +\varsigma ^2(r), \end{aligned}$$

with $\kappa$ as constant and Eq. (55) has the following solutions^34^:

**Case 1:** if $\kappa <0$, then

56 $$\begin{aligned} \varsigma (r)=-\sqrt{-\kappa } \tanh (\sqrt{-\kappa }~r), \end{aligned}$$

or

57 $$\begin{aligned} \varsigma (r)=-\sqrt{-\kappa } \coth (\sqrt{-\kappa }~r). \end{aligned}$$

**Case 2:** if $\kappa =0$, then

58 $$\begin{aligned} \varsigma (r)=-\frac{1}{r}\cdot \end{aligned}$$

**Case 3:** if $\kappa >0$, then

59 $$\begin{aligned} \varsigma (r)=\sqrt{\kappa } \tan (\sqrt{\kappa } ~r), \end{aligned}$$

or

60 $$\begin{aligned} \varsigma (r)=-\sqrt{\kappa } \cot (\sqrt{\kappa }~r). \end{aligned}$$

Putting Eq. (55) and derivatives of Eq. (54) into Eq. (30) then we get a set of algebraic equations and solve by utilizing Mathematica. The solutions we get put into Eq. (54).

## Solitary wave solutions

### Application of generalized auxiliary equation method

Applying the Homogenous balance technique on Eq. (13). Then we get $N=1$ and putting into Eq. (31).

61 $$\begin{aligned} u(r)=a_{0}+a_1 \varsigma (r). \end{aligned}$$

Putting Eq. (61) into Eq. (13), then we get following set of solutions

62 $$\begin{aligned} \alpha _0&=\frac{9 b_2^2\pm 3 \sqrt{9 b_2^4-32 b_1 b_2^2 b_3}-32 b_1 b_3}{18 b_2^2-64 b_1 b_3}, \alpha _1= \pm \frac{4 b_3 \sqrt{9 b_2^4-32 b_1 b_2^2 b_3}}{9 b_2^3-32 b_1 b_2 b_3},\\ \gamma&=\frac{9 b_2^2 d}{2 b_3}-16 b_1 d, \sigma =\frac{9 b_2^2\pm 3 \sqrt{9 b_2^4-32 b_1 b_2^2 b_3}-32 b_1 b_3}{18 b_2^2-64 b_1 b_3}. \end{aligned}$$

Now a family of solutions are

#### Hyperbolic trigonometric solutions

63 $$\begin{aligned} f(x,y)= &  \frac{9 b_2^2\pm 3 \sqrt{9 b_2^4-32 b_1 b_2^2 b_3}-32 b_1 b_3}{18 b_2^2-64 b_1 b_3}+\left( \pm \frac{4 b_3 \sqrt{9 b_2^4-32 b_1 b_2^2 b_3}}{9 b_2^3-32 b_1 b_2 b_3} \right) \left( \frac{-b_{1}b_{2}\textrm{sech} \,^{2}\left( \frac{\sqrt{b_{1}}}{2}r\right) }{b^{2}_{2}-b_{1}b_{3}\left( 1\pm \tanh \left( \frac{\sqrt{b_{1}}}{2}r\right) \right) }\right) , \end{aligned}$$

64 $$\begin{aligned} f(x,y)= &  \frac{9 b_2^2\pm 3 \sqrt{9 b_2^4-32 b_1 b_2^2 b_3}-32 b_1 b_3}{18 b_2^2-64 b_1 b_3}+\left( \pm \frac{4 b_3 \sqrt{9 b_2^4-32 b_1 b_2^2 b_3}}{9 b_2^3-32 b_1 b_2 b_3} \right) \left( \frac{b_{1}b_{2}\textrm{csch} \,^{2}\left( \frac{\sqrt{b_{1}}}{2}r\right) }{b^{2}_{2}-b_{1}b_{3}\left( 1\pm \coth \left( \frac{\sqrt{b_{1}}}{2}r\right) \right) }\right) , \end{aligned}$$

65 $$\begin{aligned} f(x,y)= &  \frac{9 b_2^2\pm 3 \sqrt{9 b_2^4-32 b_1 b_2^2 b_3}-32 b_1 b_3}{18 b_2^2-64 b_1 b_3}+\left( \pm \frac{4 b_3 \sqrt{9 b_2^4-32 b_1 b_2^2 b_3}}{9 b_2^3-32 b_1 b_2 b_3} \right) \left( \frac{2b_{1}\textrm{sech} \,^{2}\left( \sqrt{b_{1}}r\right) }{\pm \sqrt{\zeta }-b_{2}\textrm{sech} \,\left( \sqrt{b_{1}}r\right) }\right) , \end{aligned}$$

66 $$\begin{aligned} f(x,y)= &  \frac{9 b_2^2\pm 3 \sqrt{9 b_2^4-32 b_1 b_2^2 b_3}-32 b_1 b_3}{18 b_2^2-64 b_1 b_3}+\left( \pm \frac{4 b_3 \sqrt{9 b_2^4-32 b_1 b_2^2 b_3}}{9 b_2^3-32 b_1 b_2 b_3} \right) \left( \frac{2b_{1}\textrm{csch} \,^{2}\left( \sqrt{b_{1}}r\right) }{\pm \sqrt{-\zeta }-b_{2}\textrm{sech} \,\left( \sqrt{b_{1}}r\right) }\right) , \end{aligned}$$

67 $$\begin{aligned} f(x,y)= &  \frac{9 b_2^2\pm 3 \sqrt{9 b_2^4-32 b_1 b_2^2 b_3}-32 b_1 b_3}{18 b_2^2-64 b_1 b_3}+\left( \pm \frac{4 b_3 \sqrt{9 b_2^4-32 b_1 b_2^2 b_3}}{9 b_2^3-32 b_1 b_2 b_3} \right) \left( \frac{-b_{1}\textrm{sech} \,^{2}\left( \frac{\sqrt{b_{1}}}{2}r\right) }{b_{2}\pm 2\sqrt{b_{1}b_{3}}\tanh \left( \frac{\sqrt{b_{1}}}{2}r\right) }\right) , \end{aligned}$$

68 $$\begin{aligned} f(x,y)= &  \frac{9 b_2^2\pm 3 \sqrt{9 b_2^4-32 b_1 b_2^2 b_3}-32 b_1 b_3}{18 b_2^2-64 b_1 b_3}+\left( \pm \frac{4 b_3 \sqrt{9 b_2^4-32 b_1 b_2^2 b_3}}{9 b_2^3-32 b_1 b_2 b_3} \right) \left( \frac{b_{1}\textrm{csch} \,^{2}\left( \frac{\sqrt{b_{1}}}{2}r\right) }{b_{2}\pm 2\sqrt{b_{1}b_{3}}\coth \left( \frac{\sqrt{b_{1}}}{2}r\right) }\right) , \end{aligned}$$

69 $$\begin{aligned} f(x,y)= &  \frac{9 b_2^2\pm 3 \sqrt{9 b_2^4-32 b_1 b_2^2 b_3}-32 b_1 b_3}{18 b_2^2-64 b_1 b_3}+\left( \pm \frac{4 b_3 \sqrt{9 b_2^4-32 b_1 b_2^2 b_3}}{9 b_2^3-32 b_1 b_2 b_3} \right) \left( -\frac{b_{1}}{b_{2}}\left( 1\pm \tanh \left( \frac{\sqrt{b_{1}}}{2}r\right) \right) \right) , \end{aligned}$$

70 $$\begin{aligned} f(x,y)= &  \frac{9 b_2^2\pm 3 \sqrt{9 b_2^4-32 b_1 b_2^2 b_3}-32 b_1 b_3}{18 b_2^2-64 b_1 b_3}+\left( \pm \frac{4 b_3 \sqrt{9 b_2^4-32 b_1 b_2^2 b_3}}{9 b_2^3-32 b_1 b_2 b_3} \right) \left( -\frac{b_{1}}{b_{2}}\left( 1\pm \coth \left( \frac{\sqrt{b_{1}}}{2}r\right) \right) \right) . \end{aligned}$$

#### Trigonometric solutions

71 $$\begin{aligned} f(x,y)= &  \frac{9 b_2^2\pm 3 \sqrt{9 b_2^4-32 b_1 b_2^2 b_3}-32 b_1 b_3}{18 b_2^2-64 b_1 b_3}+\left( \pm \frac{4 b_3 \sqrt{9 b_2^4-32 b_1 b_2^2 b_3}}{9 b_2^3-32 b_1 b_2 b_3} \right) \left( \frac{-b_{1}\sec ^{2}\left( \frac{\sqrt{-b_{1}}}{2}r\right) }{b_{2}\pm 2\sqrt{-b_{1}b_{3}}tan\left( \frac{\sqrt{-b_{1}}}{2}r\right) }\right) , \end{aligned}$$

72 $$\begin{aligned} f(x,y)= &  \frac{9 b_2^2\pm 3 \sqrt{9 b_2^4-32 b_1 b_2^2 b_3}-32 b_1 b_3}{18 b_2^2-64 b_1 b_3}+\left( \pm \frac{4 b_3 \sqrt{9 b_2^4-32 b_1 b_2^2 b_3}}{9 b_2^3-32 b_1 b_2 b_3} \right) \left( \frac{-b_{1}\csc ^{2}\left( \frac{\sqrt{-b_{1}}}{2}r\right) }{b_{2}\pm 2\sqrt{-b_{1}b_{3}}cot\left( \frac{\sqrt{-b_{1}}}{2}r\right) }\right) , \end{aligned}$$

73 $$\begin{aligned} f(x,y)= &  \frac{9 b_2^2\pm 3 \sqrt{9 b_2^4-32 b_1 b_2^2 b_3}-32 b_1 b_3}{18 b_2^2-64 b_1 b_3}+\left( \pm \frac{4 b_3 \sqrt{9 b_2^4-32 b_1 b_2^2 b_3}}{9 b_2^3-32 b_1 b_2 b_3} \right) \left( \frac{2b_{1}\sec ^{2}\left( \sqrt{-b_{1}}r\right) }{\pm \sqrt{\zeta }-b_{2}\sec \left( \sqrt{-b_{1}}r\right) }\right) , \end{aligned}$$

74 $$\begin{aligned} f(x,y)= &  \frac{9 b_2^2\pm 3 \sqrt{9 b_2^4-32 b_1 b_2^2 b_3}-32 b_1 b_3}{18 b_2^2-64 b_1 b_3}+\left( \pm \frac{4 b_3 \sqrt{9 b_2^4-32 b_1 b_2^2 b_3}}{9 b_2^3-32 b_1 b_2 b_3} \right) \left( \frac{2b_{1}\csc ^{2}\left( \sqrt{-b_{1}}r\right) }{\pm \sqrt{\zeta }-b_{2}\sec \left( \sqrt{-b_{1}}r\right) }\right) . \end{aligned}$$

#### Exponential solutions

75 $$\begin{aligned} f(x,y)= &  \frac{9 b_2^2\pm 3 \sqrt{9 b_2^4-32 b_1 b_2^2 b_3}-32 b_1 b_3}{18 b_2^2-64 b_1 b_3}+\left( \pm \frac{4 b_3 \sqrt{9 b_2^4-32 b_1 b_2^2 b_3}}{9 b_2^3-32 b_1 b_2 b_3} \right) \left( \frac{4b_1 e^{\pm \sqrt{b_1}r}}{\left( e^{\pm \sqrt{b_1}r}-b_2\right) ^2-4b_1b_3}\right) , \end{aligned}$$

76 $$\begin{aligned} f(x,y)= &  \frac{9 b_2^2\pm 3 \sqrt{9 b_2^4-32 b_1 b_2^2 b_3}-32 b_1 b_3}{18 b_2^2-64 b_1 b_3}+\left( \pm \frac{4 b_3 \sqrt{9 b_2^4-32 b_1 b_2^2 b_3}}{9 b_2^3-32 b_1 b_2 b_3} \right) \left( \frac{\pm 4b_1 e^{\pm \sqrt{b_1}r}}{1-4b_1b_3e^{\pm 2\sqrt{b_1}r}}\right) . \end{aligned}$$

#### Rational solutions

77 $$\begin{aligned} f(x,y)= &  \frac{9 b_2^2\pm 3 \sqrt{9 b_2^4-32 b_1 b_2^2 b_3}-32 b_1 b_3}{18 b_2^2-64 b_1 b_3}+\left( \pm \frac{4 b_3 \sqrt{9 b_2^4-32 b_1 b_2^2 b_3}}{9 b_2^3-32 b_1 b_2 b_3} \right) \left( \frac{\pm b_1b_2}{b_2^2r^2-b_1b_3}\right) , \end{aligned}$$

78 $$\begin{aligned} f(x,y)= &  \frac{9 b_2^2\pm 3 \sqrt{9 b_2^4-32 b_1 b_2^2 b_3}-32 b_1 b_3}{18 b_2^2-64 b_1 b_3}+\left( \pm \frac{4 b_3 \sqrt{9 b_2^4-32 b_1 b_2^2 b_3}}{9 b_2^3-32 b_1 b_2 b_3} \right) \left( \pm \frac{1}{\sqrt{b_3}r}\right) . \end{aligned}$$

**Set-2**

79 $$\begin{aligned} &\alpha _0=1, \alpha _1=\frac{\pm \sqrt{b_2^2 \left( 9 b_2^2-32 b_1 b_3\right) d^2}+3 b_2^2 d}{4 b_1 b_2 d}, \gamma =\frac{\mp 3 \sqrt{b_2^2 \left( 9 b_2^2-32 b_1 b_3\right) d^2}+9 b_2^2 d-16 b_1 b_3 d}{4 b_3},\\ &\sigma =\frac{\mp \sqrt{b_2^2 \left( 9 b_2^2-32 b_1 b_3\right) d^2}-9 b_2^2 d+32 b_1 b_3 d}{16 b_1 b_3 d}\cdot \end{aligned}$$

Now the family of solutions are.

#### Hyperbolic trigonometric solutions

80 $$\begin{aligned} f(x,y)= &  1+\left( \frac{\pm \sqrt{b_2^2 \left( 9 b_2^2-32 b_1 b_3\right) d^2}+3 b_2^2 d}{4 b_1 b_2 d}\right) \left( \frac{-b_{1}b_{2}\textrm{sech} \,^{2}\left( \frac{\sqrt{b_{1}}}{2}r\right) }{b^{2}_{2}-b_{1}b_{3}\left( 1\pm \tanh \left( \frac{\sqrt{b_{1}}}{2}r\right) \right) }\right) , \end{aligned}$$

81 $$\begin{aligned} f(x,y)= &  1+\left( \frac{\pm \sqrt{b_2^2 \left( 9 b_2^2-32 b_1 b_3\right) d^2}+3 b_2^2 d}{4 b_1 b_2 d} \right) \left( \frac{b_{1}b_{2}\textrm{csch} \,^{2}\left( \frac{\sqrt{b_{1}}}{2}r\right) }{b^{2}_{2}-b_{1}b_{3}\left( 1\pm \coth \left( \frac{\sqrt{b_{1}}}{2}r\right) \right) }\right) , \end{aligned}$$

82 $$\begin{aligned} f(x,y)= &  1+\left( \frac{\pm \sqrt{b_2^2 \left( 9 b_2^2-32 b_1 b_3\right) d^2}+3 b_2^2 d}{4 b_1 b_2 d}\right) \left( \frac{2b_{1}\textrm{sech} \,^{2}\left( \sqrt{b_{1}}r\right) }{\pm \sqrt{\zeta }-b_{2}\textrm{sech} \,\left( \sqrt{b_{1}}r\right) }\right) , \end{aligned}$$

83 $$\begin{aligned} f(x,y)= &  1+\left( \frac{\pm \sqrt{b_2^2 \left( 9 b_2^2-32 b_1 b_3\right) d^2}+3 b_2^2 d}{4 b_1 b_2 d}\right) \left( \frac{2b_{1}\textrm{csch} \,^{2}\left( \sqrt{b_{1}}r\right) }{\pm \sqrt{-\zeta }-b_{2}\textrm{sech} \,\left( \sqrt{b_{1}}r\right) }\right) , \end{aligned}$$

84 $$\begin{aligned} f(x,y)= &  1+\left( \frac{\pm \sqrt{b_2^2 \left( 9 b_2^2-32 b_1 b_3\right) d^2}+3 b_2^2 d}{4 b_1 b_2 d} \right) \left( \frac{-b_{1}\textrm{sech} \,^{2}\left( \frac{\sqrt{b_{1}}}{2}r\right) }{b_{2}\pm 2\sqrt{b_{1}b_{3}}\tanh \left( \frac{\sqrt{b_{1}}}{2}r\right) }\right) , \end{aligned}$$

85 $$\begin{aligned} f(x,y)= &  1+\left( \frac{\pm \sqrt{b_2^2 \left( 9 b_2^2-32 b_1 b_3\right) d^2}+3 b_2^2 d}{4 b_1 b_2 d}\right) \left( \frac{b_{1}\textrm{csch} \,^{2}\left( \frac{\sqrt{b_{1}}}{2}r\right) }{b_{2}\pm 2\sqrt{b_{1}b_{3}}\coth \left( \frac{\sqrt{b_{1}}}{2}r\right) }\right) , \end{aligned}$$

86 $$\begin{aligned} f(x,y)= &  1+\left( \frac{\pm \sqrt{b_2^2 \left( 9 b_2^2-32 b_1 b_3\right) d^2}+3 b_2^2 d}{4 b_1 b_2 d}\right) \left( -\frac{b_{1}}{b_{2}}\left( 1\pm \tanh \left( \frac{\sqrt{b_{1}}}{2}r\right) \right) \right) , \end{aligned}$$

87 $$\begin{aligned} f(x,y)= &  1+\left( \frac{\pm \sqrt{b_2^2 \left( 9 b_2^2-32 b_1 b_3\right) d^2}+3 b_2^2 d}{4 b_1 b_2 d} \right) \left( -\frac{b_{1}}{b_{2}}\left( 1\pm \coth \left( \frac{\sqrt{b_{1}}}{2}r\right) \right) \right) . \end{aligned}$$

#### Trigonometric solutions

88 $$\begin{aligned} f(x,y)= &  1+\left( \frac{\pm \sqrt{b_2^2 \left( 9 b_2^2-32 b_1 b_3\right) d^2}+3 b_2^2 d}{4 b_1 b_2 d}\right) \left( \frac{-b_{1}\sec ^{2}\left( \frac{\sqrt{-b_{1}}}{2}r\right) }{b_{2}\pm 2\sqrt{-b_{1}b_{3}}tan\left( \frac{\sqrt{-b_{1}}}{2}r\right) }\right) , \end{aligned}$$

89 $$\begin{aligned} f(x,y)= &  1+\left( \frac{\pm \sqrt{b_2^2 \left( 9 b_2^2-32 b_1 b_3\right) d^2}+3 b_2^2 d}{4 b_1 b_2 d} \right) \left( \frac{-b_{1}\csc ^{2}\left( \frac{\sqrt{-b_{1}}}{2}r\right) }{b_{2}\pm 2\sqrt{-b_{1}b_{3}}cot\left( \frac{\sqrt{-b_{1}}}{2}r\right) }\right) , \end{aligned}$$

90 $$\begin{aligned} f(x,y)= &  1+\left( \frac{\pm \sqrt{b_2^2 \left( 9 b_2^2-32 b_1 b_3\right) d^2}+3 b_2^2 d}{4 b_1 b_2 d} \right) \left( \frac{2b_{1}\sec ^{2}\left( \sqrt{-b_{1}}r\right) }{\pm \sqrt{\zeta }-b_{2}\sec \left( \sqrt{-b_{1}}r\right) }\right) , \end{aligned}$$

91 $$\begin{aligned} f(x,y)= &  1+\left( \frac{\pm \sqrt{b_2^2 \left( 9 b_2^2-32 b_1 b_3\right) d^2}+3 b_2^2 d}{4 b_1 b_2 d} \right) \left( \frac{2b_{1}\csc ^{2}\left( \sqrt{-b_{1}}r\right) }{\pm \sqrt{\zeta }-b_{2}\sec \left( \sqrt{-b_{1}}r\right) }\right) . \end{aligned}$$

#### Exponential solutions

92 $$\begin{aligned} f(x,y)= &  1+\left( \frac{\pm \sqrt{b_2^2 \left( 9 b_2^2-32 b_1 b_3\right) d^2}+3 b_2^2 d}{4 b_1 b_2 d} \right) \left( \frac{4b_1 e^{\pm \sqrt{b_1}r}}{\left( e^{\pm \sqrt{b_1}r}-b_2\right) ^2-4b_1b_3}\right) , \end{aligned}$$

93 $$\begin{aligned} f(x,y)= &  1+\left( \frac{\pm \sqrt{b_2^2 \left( 9 b_2^2-32 b_1 b_3\right) d^2}+3 b_2^2 d}{4 b_1 b_2 d}\right) \left( \frac{\pm 4b_1 e^{\pm \sqrt{b_1}r}}{1-4b_1b_3e^{\pm 2\sqrt{b_1}r}}\right) . \end{aligned}$$

#### Rational solutions

94 $$\begin{aligned} f(x,y)= &  1+\left( \frac{\pm \sqrt{b_2^2 \left( 9 b_2^2-32 b_1 b_3\right) d^2}+3 b_2^2 d}{4 b_1 b_2 d}\right) \left( \frac{\pm b_1b_2}{b_2^2r^2-b_1b_3}\right) , \end{aligned}$$

95 $$\begin{aligned} f(x,y)= &  1+\left( \frac{\pm \sqrt{b_2^2 \left( 9 b_2^2-32 b_1 b_3\right) d^2}+3 b_2^2 d}{4 b_1 b_2 d}\right) \left( \pm \frac{1}{\sqrt{b_3}r}\right) . \end{aligned}$$

**Set-3**

96 $$\begin{aligned} &\alpha _0=0,\alpha _1=\frac{\pm \sqrt{b_2^2 \left( 9 b_2^2-32 b_1 b_3\right) d^2}-3 b_2^2 d}{4 b_1 b_2 d}, \gamma =\frac{\pm 3 \sqrt{b_2^2 \left( 9 b_2^2-32 b_1 b_3\right) d^2}+9 b_2^2 d-16 b_1 b_3 d}{4 b_3},\\ &\sigma =\frac{\mp 3 \sqrt{b_2^2 \left( 9 b_2^2-32 b_1 b_3\right) d^2}+9 b_2^2 d-16 b_1 b_3 d}{16 b_1 b_3 d}. \end{aligned}$$

Now a family of solutions are

#### Hyperbolic trigonometric solutions

97 $$\begin{aligned} f(x,y)= &  \left( \frac{\pm \sqrt{b_2^2 \left( 9 b_2^2-32 b_1 b_3\right) d^2}-3 b_2^2 d}{4 b_1 b_2 d}\right) \left( \frac{-b_{1}b_{2}\textrm{sech} \,^{2}\left( \frac{\sqrt{b_{1}}}{2}r\right) }{b^{2}_{2}-b_{1}b_{3}\left( 1\pm \tanh \left( \frac{\sqrt{b_{1}}}{2}r\right) \right) }\right) , \end{aligned}$$

98 $$\begin{aligned} f(x,y)= &  \left( \frac{\pm \sqrt{b_2^2 \left( 9 b_2^2-32 b_1 b_3\right) d^2}-3 b_2^2 d}{4 b_1 b_2 d} \right) \left( \frac{b_{1}b_{2}\textrm{csch} \,^{2}\left( \frac{\sqrt{b_{1}}}{2}r\right) }{b^{2}_{2}-b_{1}b_{3}\left( 1\pm \coth \left( \frac{\sqrt{b_{1}}}{2}r\right) \right) }\right) ,\end{aligned}$$

99 $$\begin{aligned} f(x,y)= &  \left( \frac{\pm \sqrt{b_2^2 \left( 9 b_2^2-32 b_1 b_3\right) d^2}-3 b_2^2 d}{4 b_1 b_2 d}\right) \left( \frac{2b_{1}\textrm{sech} \,^{2}\left( \sqrt{b_{1}}r\right) }{\pm \sqrt{\zeta }-b_{2}\textrm{sech} \,\left( \sqrt{b_{1}}r\right) }\right) ,\end{aligned}$$

100 $$\begin{aligned} f(x,y)= &  \left( \frac{\pm \sqrt{b_2^2 \left( 9 b_2^2-32 b_1 b_3\right) d^2}-3 b_2^2 d}{4 b_1 b_2 d}\right) \left( \frac{2b_{1}\textrm{csch} \,^{2}\left( \sqrt{b_{1}}r\right) }{\pm \sqrt{-\zeta }-b_{2}\textrm{sech} \,\left( \sqrt{b_{1}}r\right) }\right) ,\end{aligned}$$

101 $$\begin{aligned} f(x,y)= &  \left( \frac{\pm \sqrt{b_2^2 \left( 9 b_2^2-32 b_1 b_3\right) d^2}-3 b_2^2 d}{4 b_1 b_2 d} \right) \left( \frac{-b_{1}\textrm{sech} \,^{2}\left( \frac{\sqrt{b_{1}}}{2}r\right) }{b_{2}\pm 2\sqrt{b_{1}b_{3}}\tanh \left( \frac{\sqrt{b_{1}}}{2}r\right) }\right) ,\end{aligned}$$

102 $$\begin{aligned} f(x,y)= &  \left( \frac{\pm \sqrt{b_2^2 \left( 9 b_2^2-32 b_1 b_3\right) d^2}-3 b_2^2 d}{4 b_1 b_2 d}\right) \left( \frac{b_{1}\textrm{csch} \,^{2}\left( \frac{\sqrt{b_{1}}}{2}r\right) }{b_{2}\pm 2\sqrt{b_{1}b_{3}}\coth \left( \frac{\sqrt{b_{1}}}{2}r\right) }\right) ,\end{aligned}$$

103 $$\begin{aligned} f(x,y)= &  \left( \frac{\pm \sqrt{b_2^2 \left( 9 b_2^2-32 b_1 b_3\right) d^2}-3 b_2^2 d}{4 b_1 b_2 d}\right) \left( -\frac{b_{1}}{b_{2}}\left( 1\pm \tanh \left( \frac{\sqrt{b_{1}}}{2}r\right) \right) \right) ,\end{aligned}$$

104 $$\begin{aligned} f(x,y)= &  \left( \frac{\pm \sqrt{b_2^2 \left( 9 b_2^2-32 b_1 b_3\right) d^2}-3 b_2^2 d}{4 b_1 b_2 d} \right) \left( -\frac{b_{1}}{b_{2}}\left( 1\pm \coth \left( \frac{\sqrt{b_{1}}}{2}r\right) \right) \right) . \end{aligned}$$

#### Trigonometric solutions

105 $$\begin{aligned} f(x,y)= &  \left( \frac{\pm \sqrt{b_2^2 \left( 9 b_2^2-32 b_1 b_3\right) d^2}-3 b_2^2 d}{4 b_1 b_2 d}\right) \left( \frac{-b_{1}\sec ^{2}\left( \frac{\sqrt{-b_{1}}}{2}r\right) }{b_{2}\pm 2\sqrt{-b_{1}b_{3}}tan\left( \frac{\sqrt{-b_{1}}}{2}r\right) }\right) , \end{aligned}$$

106 $$\begin{aligned} f(x,y)= &  \left( \frac{\pm \sqrt{b_2^2 \left( 9 b_2^2-32 b_1 b_3\right) d^2}-3 b_2^2 d}{4 b_1 b_2 d} \right) \left( \frac{-b_{1}\csc ^{2}\left( \frac{\sqrt{-b_{1}}}{2}r\right) }{b_{2}\pm 2\sqrt{-b_{1}b_{3}}cot\left( \frac{\sqrt{-b_{1}}}{2}r\right) }\right) , \end{aligned}$$

107 $$\begin{aligned} f(x,y)= &  \left( \frac{\pm \sqrt{b_2^2 \left( 9 b_2^2-32 b_1 b_3\right) d^2}-3 b_2^2 d}{4 b_1 b_2 d} \right) \left( \frac{2b_{1}\sec ^{2}\left( \sqrt{-b_{1}}r\right) }{\pm \sqrt{\zeta }-b_{2}\sec \left( \sqrt{-b_{1}}r\right) }\right) , \end{aligned}$$

108 $$\begin{aligned} f(x,y)= &  \left( \frac{\pm \sqrt{b_2^2 \left( 9 b_2^2-32 b_1 b_3\right) d^2}-3 b_2^2 d}{4 b_1 b_2 d} \right) \left( \frac{2b_{1}\csc ^{2}\left( \sqrt{-b_{1}}r\right) }{\pm \sqrt{\zeta }-b_{2}\sec \left( \sqrt{-b_{1}}r\right) }\right) . \end{aligned}$$

#### Exponential solutions

109 $$\begin{aligned} f(x,y)= &  \left( \frac{\pm \sqrt{b_2^2 \left( 9 b_2^2-32 b_1 b_3\right) d^2}-3 b_2^2 d}{4 b_1 b_2 d} \right) \left( \frac{4b_1 e^{\pm \sqrt{b_1}r}}{\left( e^{\pm \sqrt{b_1}r}-b_2\right) ^2-4b_1b_3}\right) , \end{aligned}$$

110 $$\begin{aligned} f(x,y)= &  \left( \frac{\pm \sqrt{b_2^2 \left( 9 b_2^2-32 b_1 b_3\right) d^2}-3 b_2^2 d}{4 b_1 b_2 d}\right) \left( \frac{\pm 4b_1 e^{\pm \sqrt{b_1}r}}{1-4b_1b_3e^{\pm 2\sqrt{b_1}r}}\right) . \end{aligned}$$

#### Rational solutions

111 $$\begin{aligned} f(x,y)= &  \left( \frac{\pm \sqrt{b_2^2 \left( 9 b_2^2-32 b_1 b_3\right) d^2}-3 b_2^2 d}{4 b_1 b_2 d}\right) \left( \frac{\pm b_1b_2}{b_2^2r^2-b_1b_3}\right) , \end{aligned}$$

112 $$\begin{aligned} f(x,y)= &  \left( \frac{\pm \sqrt{b_2^2 \left( 9 b_2^2-32 b_1 b_3\right) d^2}-3 b_2^2 d}{4 b_1 b_2 d}\right) \left( \pm \frac{1}{\sqrt{b_3}r}\right) . \end{aligned}$$

### Application of modified $\left( \frac{G^{'}}{G^2}\right)$-expansion method

Applying the Homogenous balance technique on Eq. (13). Then we get $N=1$ and putting into Eq. (49).

113 $$\begin{aligned} U(r)=a_0+a_1\left( \frac{G^{'}}{G^2}\right) . \end{aligned}$$

Putting Eq. (113) into Eq. (15), then we get following set of solutions.

**Set-1**

114 $$\begin{aligned} a_0=\frac{\sigma +1}{2}, a_1=-\frac{2 \kappa }{\gamma \sigma +\gamma }, d=\frac{2}{\gamma (\sigma +1)^2}, \tau =-\frac{\gamma ^2 (\sigma +1)^2 \left( \sigma ^2+6 \sigma +1\right) }{16 \kappa }. \end{aligned}$$

The family of solutions are given by

**Case 1:** If $$\kappa \tau >0$$,

115 $$\begin{aligned} f(y,t)=\left( \frac{\sigma +1}{2}\right) +\left( -\frac{2 \kappa }{\gamma \sigma +\gamma }\right) \left( \sqrt{\frac{\kappa }{\tau }} \left( \frac{A_1\cos \sqrt{\kappa \tau }r+B_1\sin \sqrt{\kappa \tau }r}{A_1\sin \sqrt{\kappa \tau }r-B_1\cos \sqrt{\kappa \tau }r}\right) \right) . \end{aligned}$$

**Case 2:** If $$\kappa \tau <0$$,

116 $$\begin{aligned} f(y,t)=\left( \frac{\sigma +1}{2}\right) +\left( -\frac{2 \kappa }{\gamma \sigma +\gamma }\right) \left( -\frac{\sqrt{\mid \kappa \tau \mid }}{\tau }+\frac{\sqrt{\mid \kappa \tau \mid }}{2}\left( \frac{A_1\sinh (2\sqrt{\mid \kappa \tau \mid }r)+B_1\cosh (2\sqrt{\mid \kappa \tau \mid }r)}{A_1 \cosh (2\sqrt{\mid \kappa \tau \mid }r)+B_1\sinh (2\sqrt{\mid \kappa \tau \mid }r)}\right) \right) . \end{aligned}$$

**Case 3:** If $$\kappa =0, \tau \ne 0$$,

117 $$\begin{aligned} f(y,t)=\left( \frac{\sigma +1}{2}\right) +\left( -\frac{2 \kappa }{\gamma \sigma +\gamma }\right) \left( -\frac{A_1}{\tau (A_1r+B_1)}\right) . \end{aligned}$$

**Set-2**

118 $$\begin{aligned} &a_0=\frac{1}{4} \left( -\sqrt{\sigma ^2+6 \sigma +1}+\sigma +1\right) , a_1=\frac{\kappa \left( 3 \sqrt{\sigma ^2+6 \sigma +1}-\sigma -1\right) }{\gamma \left( 2 \sigma ^2+13 \sigma +2\right) }, \\ &d=\frac{5 \sigma ^2+\left( 28-3 \sqrt{\sigma ^2+6 \sigma +1}\right) \sigma -3 \sqrt{\sigma ^2+6 \sigma +1}+5}{\gamma \left( 2 \sigma ^2+13 \sigma +2\right) ^2},\\ &\tau =\frac{\gamma ^2 \left( -\sigma ^4+\left( 9 \sqrt{\sigma ^2+6 \sigma +1}-40\right) \sigma ^2 +3 \left( 3 \sqrt{\sigma ^2+6 \sigma +1}-4\right) \sigma \right) }{32 \kappa }\\ &+\gamma ^2\left( \frac{\sqrt{\sigma ^2+6 \sigma +1}+\left( \sqrt{\sigma ^2+6 \sigma +1}-12\right) \sigma ^3-1}{32 \kappa }\right) . \end{aligned}$$

The family of solutions are given by,

**Case 1:** If $$\kappa \tau >0$$,

119 $$\begin{aligned} f(y,t)=&\left( \frac{1}{4} \left( -\sqrt{\sigma ^2+6 \sigma +1}+\sigma +1\right) \right) +\left( \frac{\kappa \left( 3 \sqrt{\sigma ^2+6 \sigma +1}-\sigma -1\right) }{\gamma \left( 2 \sigma ^2+13 \sigma +2\right) }\right) \\ &\left( \sqrt{\frac{\kappa }{\tau }} \left( \frac{A_1\cos \sqrt{\kappa \tau }r+B_1\sin \sqrt{\kappa \tau }r}{A_1\sin \sqrt{\kappa \tau }r-B_1\cos \sqrt{\kappa \tau }r}\right) \right) . \end{aligned}$$

**Case 2:** If $$\kappa \tau <0$$,

120 $$\begin{aligned} f(y,t)=&\left( \frac{1}{4} \left( -\sqrt{\sigma ^2+6 \sigma +1}+\sigma +1\right) \right) +\left( \frac{\kappa \left( 3 \sqrt{\sigma ^2+6 \sigma +1}-\sigma -1\right) }{\gamma \left( 2 \sigma ^2+13 \sigma +2\right) }\right) \\ &\left( -\frac{\sqrt{\mid \kappa \tau \mid }}{\tau }+\frac{\sqrt{\mid \kappa \tau \mid }}{2}\left( \frac{A_1\sinh (2\sqrt{\mid \kappa \tau \mid }r)+B_1\cosh (2\sqrt{\mid \kappa \tau \mid }r)}{A_1 \cosh (2\sqrt{\mid \kappa \tau \mid }r)+B_1\sinh (2\sqrt{\mid \kappa \tau \mid }r)}\right) \right) . \end{aligned}$$

**Case 3:** If $$\kappa =0, \tau \ne 0$$,

121 $$\begin{aligned} f(y,t)=\left( \frac{1}{4} \left( -\sqrt{\sigma ^2+6 \sigma +1}+\sigma +1\right) \right) +\left( \frac{\kappa \left( 3 \sqrt{\sigma ^2+6 \sigma +1}-\sigma -1\right) }{\gamma \left( 2 \sigma ^2+13 \sigma +2\right) }\right) \left( -\frac{A_1}{\tau (A_1r+B_1)}\right) . \end{aligned}$$

### Application of extended modified $\tanh$ expansion scheme

Applying the Homogenous balance technique on Eq. (13). Then we get $N=1$ and putting into Eq. (54).

122 $$\begin{aligned} u(r)=a_0+ a_1 \varsigma (r) + b_1\varsigma ^{-1}(r). \end{aligned}$$

Putting Eq. (122) into Eq. (28), then we get the following set of solutions,

**Set-1**

123 $$\begin{aligned} a_0=\frac{\sigma +1}{2}, a_1=-2 d (\sigma +1), b_1= 0, \kappa =-\frac{\sigma ^2+6 \sigma +1}{16 d^2 (\sigma +1)^2}, \gamma =\frac{1}{d (\sigma +1)^2}, \end{aligned}$$

solution is,

124 $$\begin{aligned} f(x,t)=\left( \frac{\sigma +1}{2}\right) +\left( -2 d (\sigma +1)\right) \varsigma (r). \end{aligned}$$

Family of solution of Eq. (124) are

**Case 1:** if $\kappa <0$, then

125 $$\begin{aligned} f(x,t)= &  \left( \frac{\sigma +1}{2}\right) +\left( -2 d (\sigma +1)\right) \left( -\sqrt{-\kappa } \tanh (\sqrt{-\kappa }~r) \right) , \end{aligned}$$

126 $$\begin{aligned} f(x,t)= &  \left( \frac{\sigma +1}{2}\right) +\left( -2 d (\sigma +1)\right) \left( -\sqrt{-\kappa } \coth (\sqrt{-\kappa }~r) \right) . \end{aligned}$$

**Case 2:** if $\kappa =0$ , then

127 $$\begin{aligned} f(x,t)=\left( \frac{\sigma +1}{2}\right) +\left( -2 d (\sigma +1)\right) \left( \frac{-1}{ r} \right) . \end{aligned}$$

**Case 3:** if $\kappa >0$, then

128 $$\begin{aligned} f(x,t)= &  \left( \frac{\sigma +1}{2}\right) +\left( -2 d (\sigma +1)\right) \left( \sqrt{\kappa } \tan (\sqrt{\kappa } ~r) \right) , \end{aligned}$$

129 $$\begin{aligned} f(x,t)= &  \left( \frac{\sigma +1}{2}\right) +\left( -2 d (\sigma +1)\right) \left( -\sqrt{\kappa } \cot (\sqrt{\kappa }~r) \right) . \end{aligned}$$

**Set-2**

130 $$\begin{aligned} a_0=\frac{\sigma +1}{2}, a_1=-2 d (\sigma +1), b_1=-\frac{\sigma ^2+6 \sigma +1}{32 d \sigma +32 d}, \kappa =-\frac{\sigma ^2+6 \sigma +1}{64 d^2 (\sigma +1)^2}, \gamma =\frac{1}{d (\sigma +1)^2}\cdot \end{aligned}$$

Finally, solution is,

131 $$\begin{aligned} f(x,t)=\left( \frac{\sigma +1}{2}\right) +\left( -2 d (\sigma +1)\right) \varsigma (r)+\left( -\frac{\sigma ^2+6 \sigma +1}{32 d \sigma +32 d} \right) (\varsigma (r))^{-1}. \end{aligned}$$

Family of solution of Eq. (131) are

**Case 1:** if $\kappa <0$, then

132 $$\begin{aligned} \small f(x,t)= &  \left( \frac{\sigma +1}{2}\right) +\left( -2 d (\sigma +1)\right) \left( -\sqrt{-\kappa } \tanh (\sqrt{-\kappa }~r) \right) +\left( -\frac{\sigma ^2+6 \sigma +1}{32 d \sigma +32 d} \right) \left( -\sqrt{-\kappa } \tanh (\sqrt{-\kappa }~r) \right) ^{-1}, \end{aligned}$$

133 $$\begin{aligned} \small f(x,t)= &  \left( \frac{\sigma +1}{2}\right) +\left( -2 d (\sigma +1)\right) \left( -\sqrt{-\kappa } \coth (\sqrt{-\kappa }~r) \right) +\left( -\frac{\sigma ^2+6 \sigma +1}{32 d \sigma +32 d} \right) \left( -\sqrt{-\kappa } \coth (\sqrt{-\kappa }~r) \right) ^{-1}. \end{aligned}$$

**Case 2:** if $\kappa =0$, then

134 $$\begin{aligned} f(x,t)=\left( \frac{\sigma +1}{2}\right) +\left( -2 d (\sigma +1)\right) \left( \frac{-1}{ r} \right) +\left( -\frac{\sigma ^2+6 \sigma +1}{32 d \sigma +32 d} \right) \left( \frac{-1}{ r} \right) ^{-1}. \end{aligned}$$

**Case 3:** if $\kappa >0$, then

135 $$\begin{aligned} f(x,t)= &  \left( \frac{\sigma +1}{2}\right) +\left( -2 d (\sigma +1)\right) \left( \sqrt{\kappa } \tan (\sqrt{\kappa } ~r) \right) +\left( -\frac{\sigma ^2+6 \sigma +1}{32 d \sigma +32 d} \right) \left( \sqrt{\kappa } \tan (\sqrt{\kappa } ~r) \right) ^{-1}. \end{aligned}$$

136 $$\begin{aligned} f(x,t)= &  \left( \frac{\sigma +1}{2}\right) +\left( -2 d (\sigma +1)\right) \left( -\sqrt{\kappa } \cot (\sqrt{\kappa }~r) \right) +\left( -\frac{\sigma ^2+6 \sigma +1}{32 d \sigma +32 d} \right) \left( -\sqrt{\kappa } \cot (\sqrt{\kappa }~r) \right) ^{-1}. \end{aligned}$$

## Graphical discussion

In this section, the graphical visualization of the Biofilm model has been discussed. Iqbal *et al.*^35^ have attained the singular, bright and dark soliton by applying a new extended direct algebraic method. The physical nature of the nonlinear model is illustrated by setting suitable values to the arbitrary constants with the help of Mathematica.

**Figure 1:**
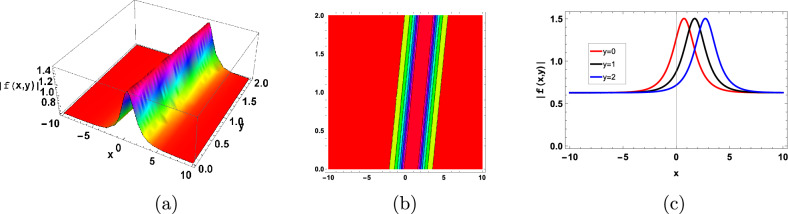
Graphical bright type soliton solution of (75, 76) with parameters $b_1=1,b_2=0.5,b_3=-1$.

**Figure 2:**
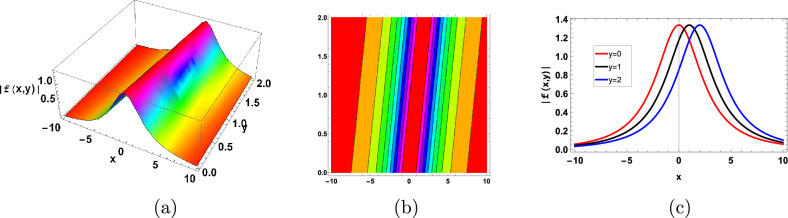
Graphical bright type soliton solution of (99) with parameters $b_1=0.01,b_2=1,b_3=-2, d=0.1$.
- Figures 3, 4 and 5 shows the anti-kink and kink soliton respectively. Kink soliton is a confined area where the biomass, metabolic activity, or microbial density abruptly change or gradient. This transition, which divides areas of distinct microbial populations or metabolic processes, usually takes place inside the biofilm across a brief spatial distance. Between the areas of high and low microbial density, there is a fixed border or interface that defines the kink soliton. The term “anti-kink soliton” refers to a specific area inside a biofilm where the normal gradient in microbial density, biomass, or metabolic activity that is seen in kink solitons is reversed or inverted.

- The Figs. 1,2 represent the bright soliton solutions. Nonlinear interactions between microbial populations, nutritional gradients, and extracellular polymeric substances (EPS) inside the biofilm matrix may be responsible for the creation and preservation of bright soliton-like patterns in biofilms. Localized patterns that resemble bright solitons can arise as a result of nonlinear factors such diffusion, microbial quorum sensing, and heterogeneity in biofilms. The behavior and ecology of biofilms may benefit from the presence of bright soliton-like structures. As an illustration, they might function as geographically restricted areas of increased microbial activity, nutrient uptake, or metabolic cooperation in the biofilm community.

**Figure 3:**
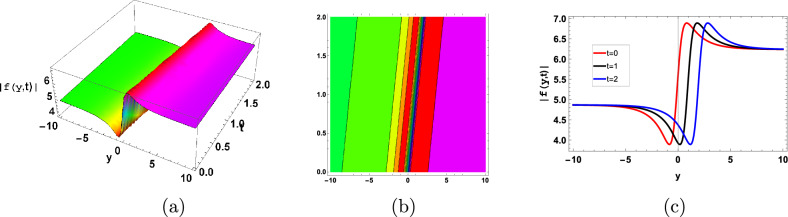
Graphical anti-kink type soliton solution of (115) with parameters $\sigma =0.8, \gamma =0.1,\kappa =-0.5, A=0.5, B=2$.

**Figure 4:**
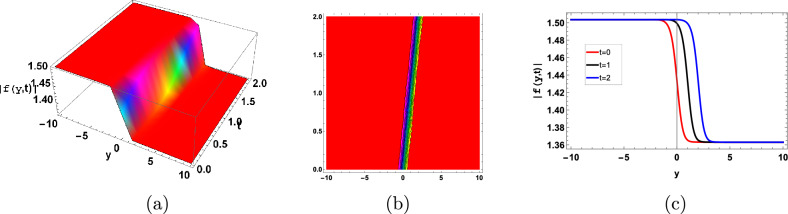
Graphical kink type soliton solution of (119) with parameters $\sigma =0.7, \gamma =0.3, \kappa =-0.1, A=2, B=0.1$.

**Figure 5:**
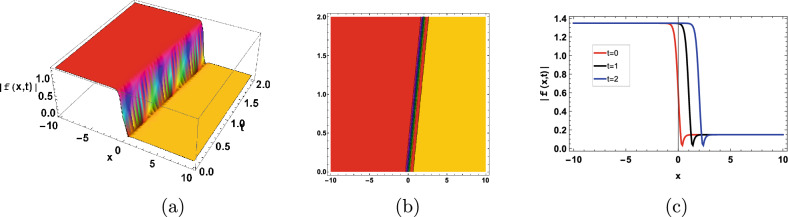
Graphical kink type soliton solution of (125) with parameters $\sigma =1, \gamma =0.3, \kappa =-1, A=1, B=0.01$.
- Figure 6 represent the dark soliton. Dark solitons impact the biofilm resilience and stability by influencing microbial dispersal, community succession, and adaptation to changing environmental conditions.

**Figure 6:**
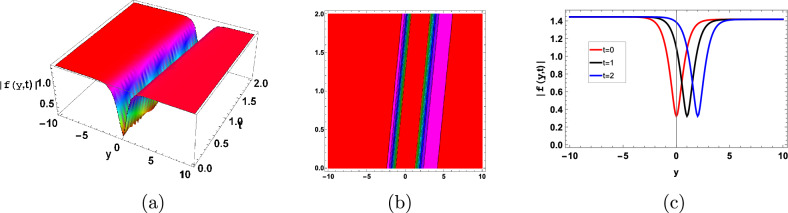
Graphical dark type soliton solution of (120) with parameters $\sigma =0.9, \gamma =0.3, \kappa =-1, A=1, B=0.01$.
- Figure 7 represent the periodic soliton solution. Periodic solitons in biofilms are frequently the result of microbial interactions, substrate availability, or cyclic environmental changes, which in turn generate spatially periodic structures within the biofilm architecture.

**Figure 7:**
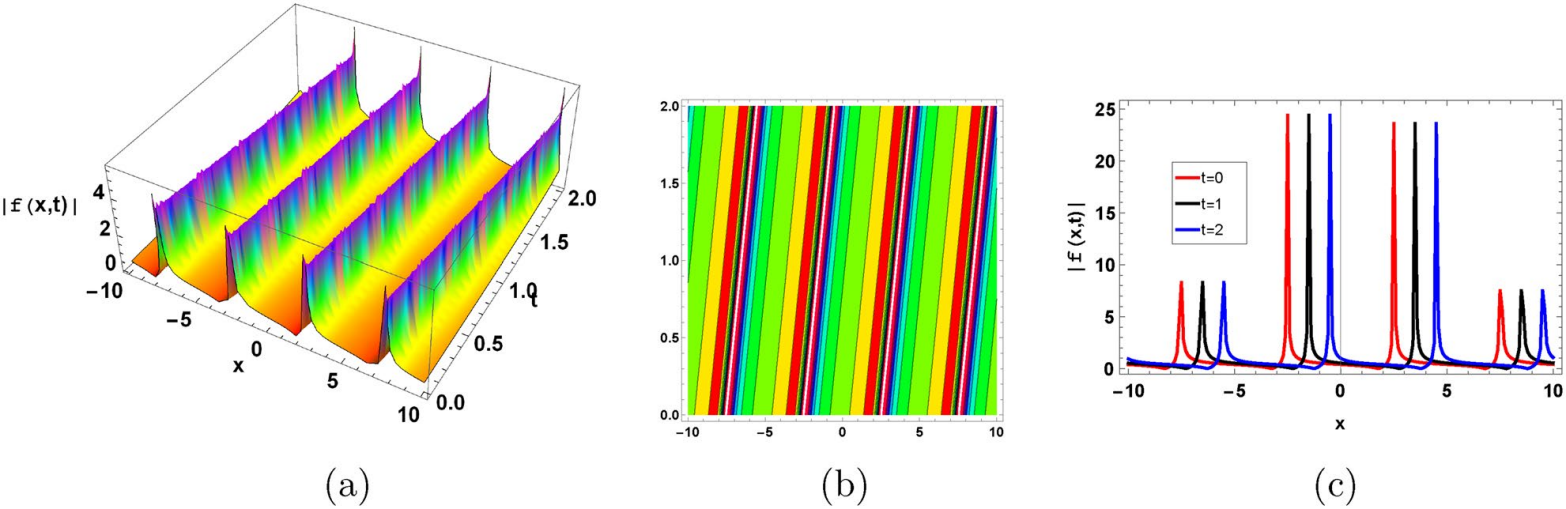
Graphical periodic type soliton solution of (128) with parameters $\sigma =0.9, \gamma =0.3, \kappa =-1, A=1, B=0.01$.

## Conclusion

In this study, we have investigated the symmetry and soliton analysis of the bistable Allen-Cahn equation with quartic potential which has applications to the biofilm model. We have applied the Lie symmetry technique on the understudy NLPDE to find out the group invariant solutions. The Lie symmetry method is employed to transform the nonlinear understudy equation into an ordinary differential equation. We have applied three analytical methods to acquire the solitary wave solutions of the considered model. To understand the physical nature of the acquired results, the 3D, 2D and contour plots are presented by considering suitable values of the involved parameters. These plots depict the singular, bright, kink, anti-kink, dark and periodic type solitons. In mathematical physics, engineering, and nonlinear sciences, it has been demonstrated that proposed methods and dynamical observations provide valuable new mathematical tools for generating precise solutions and conducting qualitative analyses in nonlinear equations (NLEs).

## Data Availability

The data sets used and/or analysed during the current study are available from the corresponding author upon reasonable request.

## References

[1] Shakeel, M. *et al.* Construction of diverse water wave structures for coupled nonlinear fractional Drinfel’d-Sokolov-Wilson model with Beta derivative and its modulus instability. *Sci. Rep.* **13**(1), 17528 (2023). 37845300 10.1038/s41598-023-44428-5 PMC10579377

[2] Shakeel, M., Bibi, A., Yasmeen, I. & Chou, D. Novel optical solitary wave structure solution of Lakshmanan-Porsezian-Daniel model. *Results Phys.* **54**, 107086 (2023).

[3] Shakeel, M., Bibi, A., AlQahtani, S. A. & Alawwad, A. M. Dynamical study of a time fractional nonlinear Schrödinger model in optical fibers. *Opt. Quant. Electron.* **55**(11), 1010 (2023).

[4] Jornet, M. Modeling of Allee effect in biofilM forMation via the stochastic bistable Allen-Cahn partial differential equation. *Stoch. Anal. Appl.* **39**(1), 22–32 (2021).

[5] Tijani, Y. O. & Appadu, A. R. Unconditionally positive NSFD and classical finite difference schemes for biofilm formation on medical implant using Allen-Cahn equation. *Demonstratio Math.* **55**(1), 40–60 (2022).

[6] Tijani, Y. O., Appadu, A. R. & Aderogba, A. A. Some finite difference methods to model biofilm growth and decay: Classical and non-standard. *Computation* **9**(11), 123 (2021).

[7] Cenesiz, Y., Tasbozan, O. & Kurt, A. Functional variable method for conformable fractional modified kdv-zkequation and Maccari system. *Tbilisi Math J.* **10**, 117–125 (2017).

[8] Zafar, A., Shakeel, M., Ali, A., Akinyemi, L. & Rezazadeh, H. Optical solitons of nonlinear complex Ginzburg-Landau equation via two modified expansion schemes. *Opt. Quant. Electron.* **54**, 1–15 (2022).

[9] Saha, D., Chatterjee, P. & Raut, S. Multi-shock and soliton solutions of the Burgers equation employing Darboux transformation with the help of the Lax pair. *Pramana* **97**(2), 54 (2023).

[10] Pankaj, R. D. Extended Jacobi elliptic function technique: A tool for solving nonlinear wave equations with emblematic software. J. Computat. Anal. Appl. 31(1) (2023).

[11] Huang, Q., Zafar, A., Raheel, M. & Bekir, A. Analytical wave solutions of an electronically and biologically important model via two efficient schemes. *Chin. Phys. B* **32**(11), 110201 (2023).

[12] Ghayad, M. S., Badra, N. M., Ahmed, H. M. & Rabie, W. B. Derivation of optical solitons and other solutions for nonlinear Schrödinger equation using modified extended direct algebraic method. *Alex. Eng. J.* **64**, 801–811 (2023).

[13] El-shamy, O., El-barkoki, R., Ahmed, H. M., Abbas, W. & Samir, I. Exploration of new solitons in optical medium with higher-order dispersive and nonlinear effects via improved modified extended tanh function method. *Alex. Eng. J.* **68**, 611–618 (2023).

[14] Caudrelier, V., Crampé, N., Ragoucy, E. & Zhang, C. Nonlinear Schrödinger equation on the half-line without a conserved number of solitons. *Physica D* **445**, 133650 (2023).

[15] Günhan Ay, N. & Yaşar, E. Novel dispersive soliton solutions to a fractional nonlinear Schrödinger equation related with ultrashort pulses. *Pramana J. Phys.* **97**, 106 (2023).

[16] Canzian, E. P., Santiago, F., Lopes, A. B., Barbosa, M. R. & Barañano, A. G. On the application of the double integral method with quadratic temperature profile for spherical solidification of lead and tin metals. *Appl. Therm. Eng.* **219**, 119528 (2023).

[17] Biswas, A. *et al.* Optical solitons in nano-fibers with spatio-temporal dispersion by trial solution method. *Optik* **127**(18), 7250–7257 (2016).

[18] Zafar, A., Shakeel, M., Ali, A., Rezazadeh, H. & Bekir, A. Analytical study of complex Ginzburg-Landau equation arising in nonlinear optics. *J. Nonlinear Opt. Phys. Mater.* **32**(01), 2350010 (2023).

[19] Kumar, S. & Niwas, M. Exploring lump soliton solutions and wave interactions using new Inverse -expansion approach: applications to the (2+ 1)-dimensional nonlinear Heisenberg ferromagnetic spin chain equation. *Nonlinear Dyn.* **111**(21), 20257–20273 (2023).

[20] Niwas, M. & Kumar, S. Multi-peakons, lumps, and other solitons solutions for the (2+ 1)-dimensional generalized Benjamin-Ono equation: an inverse (/G)-expansion method and real-world applications. *Nonlinear Dyn.* **111**(24), 22499–22512 (2023).

[21] Zafar, A., Inc, M., Shakeel, M. & Mohsin, M. Analytical study of nonlinear water wave equations for their fractional solution structures. *Mod. Phys. Lett. B* **36**(14), 2250071 (2022).

[22] Riaz, M. B., Baleanu, D., Jhangeer, A. & Abbas, N. Nonlinear self-adjointness, conserved vectors, and traveling wave structures for the kinetics of phase separation dependent on ternary alloys in iron (Fe-Cr-Y (Y= Mo, Cu)). *Results Phys.* **25**, 104151 (2021).

[23] Kumar, S. & Niwas, M. Analyzing multi-peak and lump solutions of the variable-coefficient Boiti-Leon-Manna-Pempinelli equation: a comparative study of the Lie classical method and unified method with applications. *Nonlinear Dyn.* **111**(24), 22457–22475 (2023).

[24] Raza, N. & Rafiq, M. H. Abundant fractional solitons to the coupled nonlinear Schrodinger equations arising in shallow water waves. *Int. J. Mod. Phys. B* **34**(18), 2050162 (2020).

[25] Kumar, S. & Dhiman, S. K. Exploring cone-shaped solitons, breather, and lump-forms solutions using the lie symmetry method and unified approach to a coupled breaking soliton model. *Phys. Scr.* **99**(2), 025243 (2024).

[26] Kumar, S., Kumar, D. & Kumar, A. Lie symmetry analysis for obtaining the abundant exact solutions, optimal system and dynamics of solitons for a higher-dimensional Fokas equation. *Chaos Solitons Fractals* **142**, 110507 (2021).

[27] Kumar, S., Ma, W. X. & Kumar, A. Lie symmetries, optimal system and group-invariant solutions of the (3+ 1)-dimensional generalized KP equation. *Chin. J. Phys.* **69**, 1–23 (2021).

[28] Kumar, S., Ma, W. X., Dhiman, S. K. & Chauhan, A. Lie group analysis with the optimal system, generalized invariant solutions, and an enormous variety of different wave profiles for the higher-dimensional modified dispersive water wave system of equations. *Eur. Phys. J. Plus* **138**(5), 434 (2023).

[29] Kumar, S., Kumar, D. & Wazwaz, A. M. Lie symmetries, optimal system, group-invariant solutions and dynamical behaviors of solitary wave solutions for a (3+ 1)-dimensional KdV-type equation. *Eur. Phys. J. Plus* **136**(5), 531 (2021).

[30] Bluman, G. W. & Cole, J. D. The general similarity solution of the heat equation. *J. Math. Mech.* **18**(11), 1025–1042 (1969).

[31] Cimpoiasu, R. & Constantinescu, R. Invariant solutions of the Eckhaus-Kundu model with nonlinear dispersion and non-Kerr nonlinearities. *Waves Random Complex Media* **31**, 331–341 (2021).

[32] Cimpoiasu, R. Multiple invariant solutions of the 3 D potential Yu-Toda-Sasa-Fukuyama equation via symmetry technique. *Int. J. Mod. Phys. B* **34**(20), 2050188 (2020).

[33] Shakeel, M., Bibi, A., Chou, D. & Zafar, A. Study of optical solitons for Kudryashov’s Quintuple power-law with dual form of nonlinearity using two modified techniques. *Optik* **273**, 170364 (2023).

[34] Raslan, K. R., Ali, K. K. & Shallal, M. A. The modified extended tanh method with the Riccati equation for solving the space-time fractional EW and MEW equations. *Chaos Solitons Fractals* **103**, 404–409 (2017).

[35] Iqbal, M. S., Sohail, S., Khurshid, H. & Chishti, K. Analysis and soliton solutions of biofilm model by new extended direct algebraic method. *Nonlinear Anal. Modell. Control* **28**, 1–16 (2023).

[36] PerthaMe, B. & PerthaMe, B. *Parabolic Equations in Biology* 1–21 (Springer International Publishing, 2015).

